# Viewpoint: a multidisciplinary approach to the assessment of patients with systemic sclerosis-associated interstitial lung disease

**DOI:** 10.1007/s10067-022-06408-4

**Published:** 2022-10-21

**Authors:** Soumya Chatterjee, Apostolos Perelas, Ruchi Yadav, Donald F. Kirby, Amandeep Singh

**Affiliations:** 1 Department of Rheumatic and Immunologic Diseases, Orthopedic and Rheumatology Institute, Cleveland Clinic, Cleveland, OH USA; 2 Department of Pulmonary and Critical Care Medicine, Virginia Commonwealth University, Richmond, VA USA; 3 Department of Diagnostic Radiology, Imaging Institute, Cleveland Clinic, Cleveland, OH USA; 4 Department of Gastroenterology, Hepatology, and Nutrition, Center for Human Nutrition, Digestive Disease and Surgery Institute, Cleveland Clinic, Cleveland, OH USA

**Keywords:** Diagnosis, Pulmonary fibrosis, Screening, Systemic sclerosis, Tomography

## Abstract

Systemic sclerosis (SSc) is a rare and heterogeneous disease affecting the skin and internal organs. SSc-associated ILD (SSc-ILD) is a common and often early manifestation of SSc. This article discusses the rationale for a multidisciplinary approach (MDA) to the early identification and assessment of patients with SSc-ILD. Diagnosis of SSc-ILD is often challenging as patients with early disease can be asymptomatic, and SSc-ILD symptoms, such as exertional dyspnea and cough, are non-specific. High-resolution computed tomography (HRCT) of the lungs is the gold standard for diagnosis of SSc-ILD since pulmonary function tests lack sensitivity and specificity, especially in early disease. On HRCT, most patients with SSc-ILD have a non-specific interstitial pneumonia (NSIP) pattern. In addition, findings of pulmonary hypertension and esophageal dysmotility may be present. The multi-organ involvement of SSc and the diverse spectrum of symptoms support an MDA for the diagnosis and assessment of patients with SSc-ILD, with input from rheumatologists, pulmonologists, gastroenterologists, radiologists, and other specialists.

**Table:** 

**Key Points** • *Interstitial lung disease (ILD) is a common manifestation of systemic sclerosis (SSc).* • *Early diagnosis is key to reducing the morbidity and mortality associated with SSc-ILD and other manifestations of SSc.* • *The multi-organ involvement of SSc supports a multidisciplinary approach to the diagnosis and assessment of patients with SSc-ILD, with input from rheumatologists, pulmonologists, gastroenterologists, radiologists, and other specialists.*

**Supplementary Information:**

The online version contains supplementary material available at 10.1007/s10067-022-06408-4.

## Introduction

Systemic sclerosis (SSc) is a rare autoimmune rheumatologic disease characterized by inflammation, fibrosis of the skin and internal organs, and vasculopathy [1]. SSc has an estimated prevalence rate of 3–24 per 100,000 [2]. Patients with SSc have an increased mortality rate. A recent study in France reported a standardized mortality ratio (SMR) of 5.73 among patients with SSc, with 28% dying within ten years of diagnosis [3]. Previous studies reported SMRs between 2.03 and 4.06 [4–6]. Pulmonary complications, i.e., pulmonary hypertension (PH) and interstitial lung disease (ILD), are the leading causes of death in patients with SSc [7, 8]. The former occurs in about 10% of patients with SSc and results from vascular remodeling, while the latter results from chronic inflammation and scarring of the lung parenchyma [9]. Data from the EUSTAR database suggest that ILD may account for 17% of SSc-related mortality [10].

Depending on the cohort and the definition used, the prevalence of ILD in SSc may be as high as 52.3%, and although it can occur at any time, it is usually an early manifestation of SSc [11–14]. Clinical and laboratory risk factors that can predict the development of SSc-ILD have been described. Patients with diffuse cutaneous systemic sclerosis (dcSSc) and positivity for anti-topoisomerase antibody (ATA) are more likely to develop ILD. However, patients with limited cutaneous SSc (lcSSc) and negativity for ATA may still develop ILD [12, 13, 15]. The anti-centromere antibody, which tends to be associated with lcSSc, is associated with a lower likelihood of developing ILD [12, 13].

The course of SSc-ILD is variable [16]. Although risk factors for the progression of SSc-ILD have been identified, such as a greater extent of fibrosis on HRCT and lower FVC, the course of SSc-ILD in an individual patient remains unpredictable [17, 18]. Several biomarkers have been investigated as predictors for the development or outcomes of SSc-ILD, including cytokines (e.g., IL-6), chemokine ligands (CCL18, CCL2, CXCL4), matrix enzymes (matrix metalloproteinases 7 and 12), secretory products of pneumocytes (KL-6), and anti-Ro52 [10, 19–25]; however, none is widely available, perhaps with the exception of KL-6, which has been approved for clinical use in Japan.

Early diagnosis is key to reducing the morbidity and mortality associated with SSc-ILD. We believe that similar to what has been described for other types of ILD [26–29], a multidisciplinary approach (MDA) can improve the outcomes of patients with SSc-ILD through timely detection and initiation/escalation of therapy. This incorporates clinical, laboratory, and imaging data, with the potential contribution of serum biomarkers, and requires real-time interaction of different specialists to establish a comprehensive co-management model that can potentially change the course of the disease.

In this article, we aim to discuss the rationale for an MDA for diagnosing and assessing patients with SSc-ILD, focusing on the role of various clinical specialists, including rheumatologists, pulmonologists, gastroenterologists, and radiologists.

## SSc-ILD: clinical features

### Patient presentation and referral

Being a systemic disorder, SSc has a heterogeneous range of manifestations: the lungs, kidneys, heart, and gastrointestinal tract may all be affected, complicating disease management. Manifestations of SSc may include skin thickening, Raynaud’s phenomenon, fingertip lesions, secondary Sjögren syndrome, musculoskeletal manifestations, gastrointestinal problems, and lung disease [30]. If pulmonary symptoms (such as shortness of breath or a chronic dry cough) are subtle or absent on initial presentation, then it is often a rheumatologist who will assess whether there is lung involvement and, if considered necessary, refer the patient to a pulmonologist for further assessment. If the patient has pulmonary symptoms, crackles on auscultation, lung abnormalities on a high-resolution computed tomography (HRCT) scan, and/or impaired lung function on pulmonary function tests, the patient is often referred to a pulmonologist. It is important to note that even in combination with a chest X-ray, the physical examination has sub-optimal sensitivity to detect SSc-ILD, especially in the early stages, and should not be used as a screening strategy [31].

### Physical and functional examination

Patients with SSc-ILD commonly present with exertional dyspnea and cough, but some patients with early SSc-ILD are asymptomatic [32, 33]. As the extent of pulmonary involvement increases, patients usually report fatigue and dyspnea on exertion and eventually at rest. In addition, chest auscultation may reveal dry crackles at the bases of the lungs [1].

Spirometry is non-invasive and cost-effective but has limitations in the setting of SSc-ILD. Assessment of forced vital capacity (FVC) lacks sensitivity, particularly in the early stages of the disease, given the wide range of normal values [34]. Moreover, it may be falsely normal in patients with coexisting emphysema [35] or falsely reduced in patients with extrapulmonary restriction [36]. The diffusing capacity for carbon monoxide (DLCO) may also be reduced, usually in proportion to the reduction in FVC. A disproportionate reduction in DLCO should raise suspicion for PH.

An assessment of functional capacity should be performed during SSc-ILD diagnosis. Although a comprehensive test such as a cardiopulmonary exercise test can provide excellent information on functional status and help predict patient outcomes [37], it may not be feasible due to resource limitations. However, a simple 6-min walk test can be performed in the office [38].

Resting and walking oximetry should be considered in patients with respiratory symptoms or abnormalities in imaging or spirometry. However, the reliability of finger oximetry is compromised in patients with poor peripheral circulation or Raynaud’s phenomenon [39]. Therefore, an alternative site, such as the earlobe or a forehead probe, should be considered.

### Radiological features of SSc-ILD

**Fig. 1:**
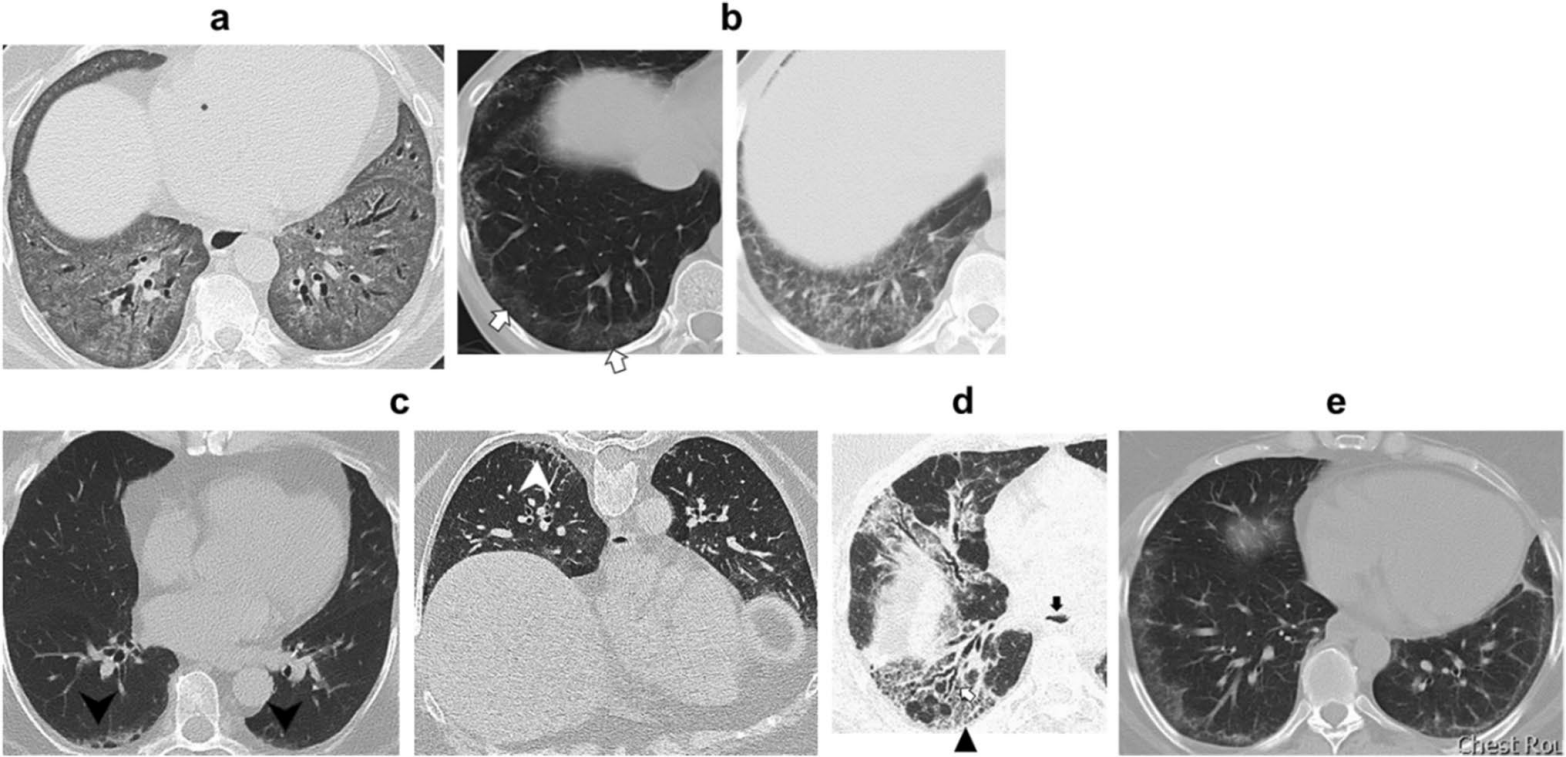
**a** Chest CT scan of a 52-year-old female with SSc demonstrating lower lobe–predominant diffuse ground-glass opacities suggestive of NSIP imaging pattern. **b** Unenhanced axial chest CT images of two patients with SSc-ILD showing lower lobe–predominant ground-glass opacity, the dominant feature of NSIP. In the image on the left, ground-glass opacities in the lower lobes are peripheral in distribution (white arrows); these are more diffuse in the image on the right. **c** Dependent opacities (black arrowheads) in the lung bases of a patient with SSc that persist on the right on the prone image (white arrowhead), suggesting early interstitial fibrosis. **d** Unenhanced axial chest CT image of a 57-year-old female with SSc-ILD showing ground-glass opacities and reticular changes in a peribronchovascular (white arrowhead) and subpleural (black arrowhead) distribution in the lower lobe. The black arrow indicates a dilated esophagus. **e** Chest CT scan showing subpleural sparing. CT, computed tomography; NSIP, non-specific interstitial pneumonia; SSc-ILD, systemic sclerosis-associated interstitial lung disease

**Fig. 2:**
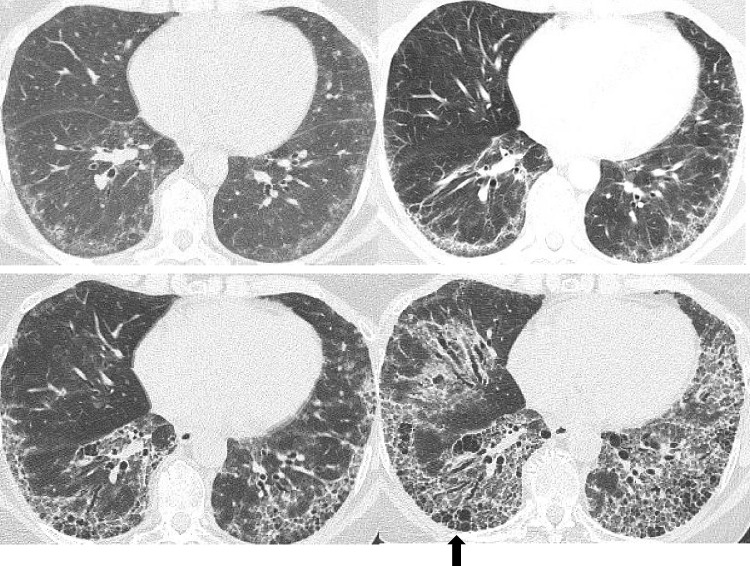
Serial chest HRCT scans of a 57-year-old female with SSc over the course of 8 years demonstrate the progression of interstitial fibrosis to such an extent that despite the initial HRCT (top left) demonstrating findings highly suggestive of NSIP, the most recent imaging (bottom right) shows advanced fibrotic ILD and honeycombing (black arrow) and looks more like UIP. HRCT, high-resolution computed tomography; NSIP, non-specific interstitial pneumonia; ILD, interstitial lung disease; UIP, usual interstitial pneumonia

**Fig. 3:**
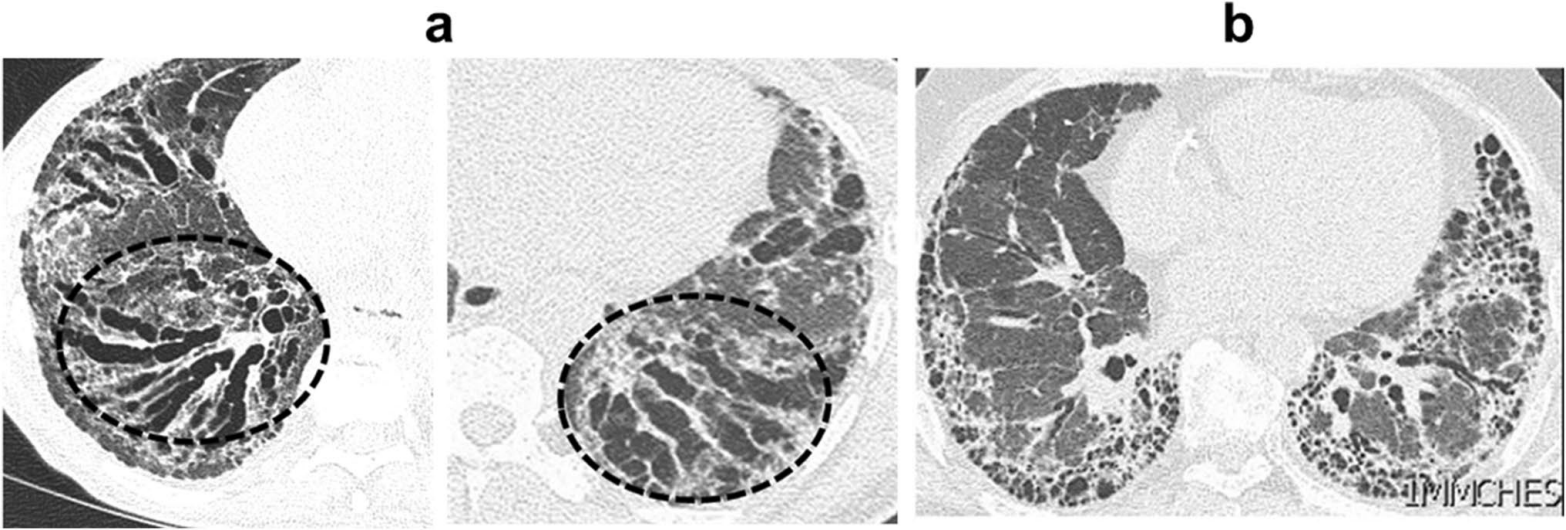
**a** Unenhanced axial chest CT images of two patients with SSc-ILD showing central traction bronchiectasis (highlighted within the black circles). **b** Chest HRCT scan of a patient with SSc-ILD and a peripheral usual interstitial pneumonia pattern of fibrosis**.** CT, computed tomography; HRCT, high-resolution computed tomography**;** SSc-ILD, systemic sclerosis-associated interstitial lung disease

HRCT is the gold standard for diagnosing SSc-ILD. On HRCT, most patients with SSc-ILD have non-specific interstitial pneumonia (NSIP) pattern, while a usual interstitial pneumonia (UIP) pattern is found in a minority (7.5%) of cases [40]. Ground-glass opacity (GGO) is the dominant feature of NSIP (Fig. 1a). It is usually symmetric in distribution, primarily subpleural, but can be diffuse (Fig. 1b), with a lower lobe predominance. During the early stages of SSc-ILD, prone images help differentiate between an increased density due to ILD and gravity-dependent atelectasis at the posterior lung bases (Fig. 1c). In patients with fibrotic NSIP, peribronchovascular and subpleural reticular opacities are found in the lower lobes (Fig. 1d). Honeycomb cystic changes can be detected in up to a third of patients with SSc-ILD [41]. The most specific sign of the NSIP pattern is subpleural sparing (Fig. 1e) [42]. In up to two-thirds of patients with SSc-ILD, GGO evolves into coarse reticular changes and traction bronchiectasis/traction bronchiolectasis (Fig. 2) [43]. The traction bronchiectasis in NSIP tends to be more central versus peripheral in UIP (Fig. 3a). The UIP pattern is characterized by basilar (occasionally diffuse) and subpleural predominant coarse reticular abnormalities, peripheral traction bronchiectasis and honeycombing (Fig. 3b) [44]. GGO occurs in a minority (15%) of patients with UIP [45].

The presence of the “straight-edge” sign (Supplementary Fig. 1a), “exuberant honeycombing” sign (Supplementary Fig. 1b), or “anterior upper lobe” sign (Supplementary Fig. 1c) increases the likelihood of connective tissue disease-associated ILD (CTD-ILD) as opposed to idiopathic pulmonary fibrosis (IPF) [44]. Multi-compartment involvement in HRCT scans suggests an underlying CTD. Esophageal dilatation on HRCT scans is predictive of an SSc diagnosis [46]. A dilated or patulous esophagus containing fluid, gas, and/or debris indicates esophageal dysmotility (Supplementary Fig. 2) [47]. Esophageal dysmotility predisposes patients to pulmonary aspiration [48], which presents with findings of bronchopneumonia and bronchiolitis in the posterior lung on CT (Supplementary Fig. 3) [49]. Associations between esophageal diameter and ILD severity may indicate that repetitive micro-aspiration of stomach content into the lungs can increase the risk of ILD progression [50].

An increased diameter of the main pulmonary artery (MPA), or the ratio of the MPA diameter relative to that of the ascending aorta (Supplementary Fig. 4), indicates PH [51]. A high MPA diameter and a segmental artery-to-bronchus diameter ratio above 1 in three to four pulmonary lobes have very high specificity for PH [51]. Pulmonary veno-occlusive disease (PVOD) is an essential consideration in SSc because PVOD-like involvement has been associated with a worse prognosis [52].

Despite the central role of HRCT in diagnosing and assessing SSc-ILD, there are concerns about potential harm associated with radiation exposure. The last decade has seen an evolution of imaging protocols and procedures to lower the radiation dose. The HRCT slice number can be reduced without jeopardizing the assessment of lung fibrosis (severity or extent) [53, 54].

### Pathologic features in SSc-ILD

Pathology is rarely, if ever, required to establish a diagnosis of SSc-ILD. Given the morbidity and mortality associated with the procedure [55], surgical lung biopsy should only be performed in exceptional cases, e.g., to exclude malignancy or where there is a suspicion of PVOD. When a biopsy is obtained, the microscopic patterns reflect the imaging. The most common pattern is NSIP, observed in 64–77% of patients [56, 57]. There is no evidence that the pattern on a biopsy has any prognostic value [56].

### Gastrointestinal manifestations of SSc-ILD

Up to 90% of patients with SSc have GI tract involvement [58]. The entire GI tract can be affected, but the most common sites are the esophagus (80%), small intestine (40–90%), and stomach (20–50%) [59].

Diagnosing and managing GI manifestations of SSc, such as gastroesophageal reflux disease (GERD) and dysmotility from an early stage, is of paramount importance, as they may lead to significant morbidity [60]. A gastroenterologist with expertise in GI dysmotility should evaluate all patients with SSc early in the disease course. Patients with SSc-ILD have higher esophageal acid exposure and higher numbers of reflux episodes than patients with SSc without ILD [61]. A modified barium swallow can help rule out oropharyngeal dysphagia in patients with swallowing issues [58]. Upper endoscopy is indicated in patients with dysphagia to identify GERD/esophagitis [62]. Patients with SSc with severe esophageal dysmotility appear to have lower DLCO and a higher prevalence of ILD [63]. Esophageal manometry should be considered to determine the severity of esophageal dysmotility in patients with esophageal dysphagia.

## Screening for and monitoring SSc-ILD

The high prevalence of ILD among patients with SSc, and the significant morbidity and mortality associated with SSc-ILD, make it essential to screen for SSc-ILD to improve patient outcomes. Different approaches exist; however, it is obvious that some type of advanced imaging is necessary, given the disappointing results of an approach based solely on physical examination, chest radiography, and pulmonary function testing [31, 34]. Protocols based on a limited number of slices seem to perform similarly to traditional HRCTs and are associated with radiation exposure similar to a plain chest X-ray (0.05 mSv) [53]. The advent of lung ultrasound has provided another tool to investigate the lung in SSc. Although ultrasonography is operator-dependent, and its validity and reliability in detecting SSc-ILD require further investigation [64], it performs well in the hands of experienced operators [65]. Its value is expected to increase as more pulmonology trainees graduate from their training programs proficient in this modality.

In a Delphi consensus study, experts in the field of SSc-ILD recommended that all patients with SSc should be screened for ILD with HRCT as the primary tool, supported by auscultation, pulmonary function testing, and assessment of symptoms [66]. Other expert groups have suggested a similar approach [30, 66–69]. In addition, all patients with SSc without a diagnosis of ILD should be screened regularly with pulmonary function tests at a frequency guided by risk assessment [66]. Spirometry and DLCO should be assessed every 3–6 months for the first 3–5 years after diagnosis of SSc when the risk of developing ILD is most significant [67].

Patients with SSc-ILD should be monitored closely to ascertain progression. Unfortunately, there is no established protocol for monitoring patients with SSc-ILD. Therefore, a multifaceted approach is required [66]. It is generally recommended that symptoms, FVC and DLCO, and exercise-induced oxygen desaturation be assessed approximately every 6–12 months, with repeat HRCT scans as clinically indicated [30, 66, 68–70]. The information gained from these assessments should guide decisions about treatment initiation, change, or escalation [66].

## Advantages of an MDA in the assessment of patients with SSc-ILD

Diagnosis and assessment of SSc-ILD, based on evaluation of clinical data, lung function tests, and imaging, requires collaboration among, at a minimum, a rheumatologist, a pulmonologist, and a radiologist. Assessment of the severity of ILD and risk factors for progression can have important implications for the patient’s prognosis, monitoring, and management [66]. Close collaboration between rheumatology and pulmonology helps personalize disease management and may result in earlier referrals for therapies such as hematopoietic stem cell transplantation and lung transplantation. Given the systemic nature of SSc, an MDA is also essential for assessing other organ manifestations. A gastroenterologist should evaluate all patients with SSc early in the disease course. Patients with SSc and potential cardiac involvement should be evaluated by a cardiologist [71]. An MDA approach is necessary to ensure a holistic approach to care, which is valued by patients [72, 73]. A summary of our recommendations is provided in Table 1.

**Table 1:** Recommendations for a multidisciplinary approach to the diagnosis and assessment of patients with SSc-ILD

• Screen all patients with SSc for ILD using HRCT
• Assess the following in patients with SSc-ILD: - The extent of fibrosis on HRCT - Respiratory symptoms - FVC - DLco - Exercise capacity (e.g., 6-min walk test) - Screen for pulmonary hypertension - Consider resting and walking oximetry
• Assess extrapulmonary manifestations of SSc: - Perform a comprehensive assessment of rheumatic manifestations - Involve a gastroenterologist in the evaluation of gastrointestinal issues such as esophageal dysmotility - Refer patients with cardiac involvement to a cardiologist
• Monitor progression of SSc-ILD approximately every 6 months using multiple methods: - Respiratory symptoms - Pulmonary function tests - Exercise-induced oxygen desaturation • Repeat HRCT should be performed as clinically indicated
• Discuss all information collected during monitoring in an MDT to develop an individualized and holistic management plan for the patient - Consider both pharmacological and non-pharmacological treatment options

*DLco*, diffusing capacity for carbon monoxide; *FVC*, forced vital capacity; *HRCT*, high-resolution computed tomography; *MDT,* multidisciplinary team; *SSc-ILD*, systemic sclerosis-associated interstitial lung disease

## Conclusions

We believe that establishing an MDA as a gold standard for diagnosing and assessing patients with SSc-ILD is advantageous for both patients and clinicians. Clinicians across specialties benefit from a better understanding of the significance of lung abnormalities in patients with SSc. The course of SSc-ILD is unpredictable, and patients should be monitored regularly to assess disease progression. An MDA is valuable to consider the totality of the information obtained from monitoring and agree on how that individual patient should be managed. Working together encourages cross-collaboration among clinical specialties in assessing and managing patients with SSc-ILD, which would ultimately benefit patients. We suggest that the multidisciplinary team should include, at a minimum, a rheumatologist, a pulmonologist, a thoracic radiologist, and a gastroenterologist.

A case study describing the challenges of managing a patient with multiple manifestations of SSc is provided in the supplementary material.

## Supplementary Information

Below is the link to the electronic supplementary material.

Supplementary file1 (DOCX 2.20 mb)

## Data Availability

No further data are available.
