# Supplementary material for: Viewpoint: a multidisciplinary approach to the assessment of patients with systemic sclerosis-associated interstitial lung disease
Source: Clin Rheumatol. 2022 Oct 21;42(3):653–61. doi: 10.1007/s10067-022-06408-4 (PMC9935731; doi:10.1007/s10067-022-06408-4)

**Supplementary information**

**A multidisciplinary approach to the assessment of patients with systemic sclerosis-associated interstitial lung disease**

Soumya Chatterjee, MD^1^, Apostolos Perelas, MD^2^, Ruchi Yadav, MD^3^, Donald F. Kirby, MD^4^, and Amandeep Singh, MD^4^

^1^Department of Rheumatic and Immunologic Diseases, Cleveland Clinic, Cleveland, Ohio, USA; ^2^Department of Pulmonary and Critical Care Medicine, Virginia Commonwealth University, Richmond, Virginia, USA; ^3^Department of Diagnostic Radiology, Imaging Institute, Cleveland Clinic, Cleveland, Ohio, USA; ^4^Department of Gastroenterology, Hepatology, and Nutrition; Center for Human Nutrition, Digestive Disease and Surgery Institute, Cleveland Clinic, Cleveland, Ohio, USA.

**Corresponding author:** Soumya Chatterjee, Department of Rheumatic and Immunologic Diseases, Cleveland Clinic, Cleveland, Ohio, USA. Email: [CHATTES@ccf.org](mailto:CHATTES@ccf.org). Tel: 001 216 444 9945

**Supplementary Figure 1.** Straight-edge, exuberant honeycombing, and anterior upper lobe signs in connective tissue disease-ILD: **a)** Unenhanced coronal chest CT image of a 54-year-old female with basilar predominant fibrotic ILD and honeycombing, illustrating the straight-edge sign. There is a sharp demarcation of the fibrosis in the craniocaudal plane without significant extension along the lateral margins of the lungs. **b)** Unenhanced axial chest CT image of a 65-year-old female with SSc-ILD, demonstrating the exuberant honeycombing sign ‒ basilar predominant pulmonary fibrosis characterized primarily by florid honeycombing (white arrows). The black arrow indicates the dilated esophagus. **c)** Unenhanced axial chest CT image of a 45-year-old female with SSc-ILD with subpleural honeycombing within the anterior upper lobes, worse on the right than on the left (white arrows), illustrating the anterior upper-lobe sign. The black arrow indicates the dilated esophagus. CT, computed tomography; ILD, interstitial lung disease; SSc-ILD, systemic sclerosis-associated interstitial lung disease.

**
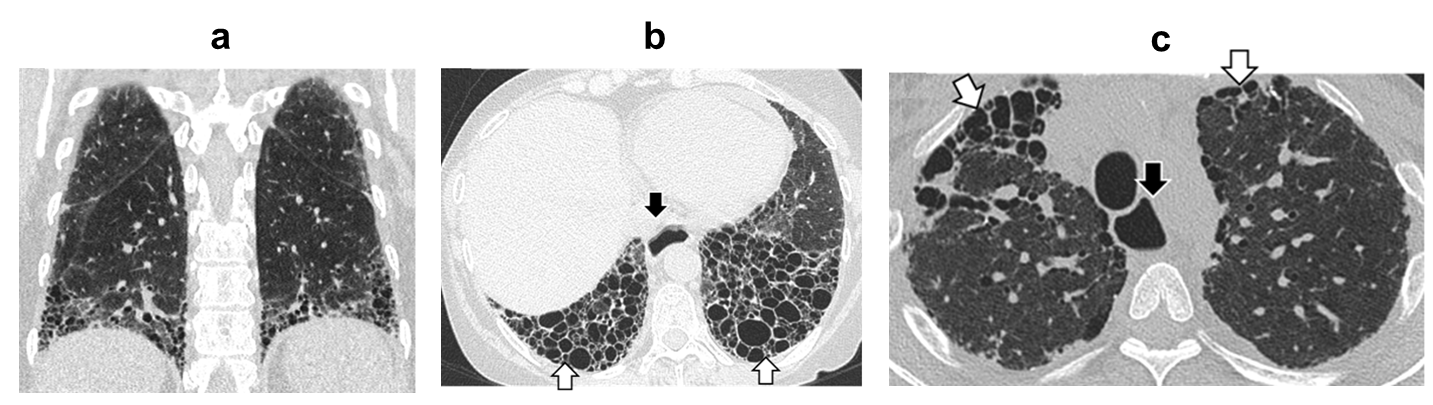
**

**Supplementary Figure 2.** Unenhanced axial chest CT image of a 52-year-old female with SSc-ILD. The black arrow indicates a dilated esophagus. The white arrows indicate subpleural sparing of the lungs immediately adjacent to the pleura in the dorsal aspect of the lower lobes. CT, computed tomography; SSc-ILD, systemic sclerosis-associated interstitial lung disease.

**
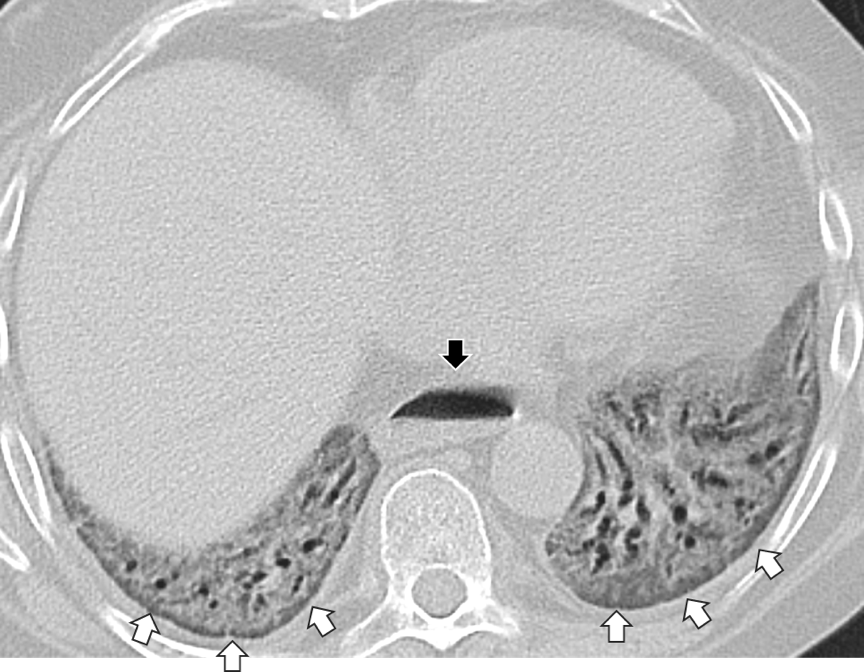
**

**Supplementary Figure 3.** Chest CT of a 56-year-old female with SSc who presented with fever, demonstrating a dilated esophagus with air-fluid level (black arrow), diffuse micronodules (bronchiolitis), and peribronchial nodular consolidation (dashed circle) in the dependent right lower lobe, findings related to aspiration pneumonia. CT, computed tomography; SSc, systemic sclerosis.


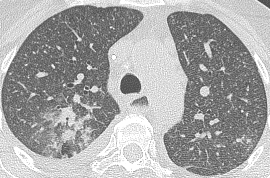


**Supplementary Figure 4.** Enlarged MPA in a patient with SSc. The CT image shows that the MPA calibre is enlarged (greater than 2.9 cm). The main PA to the ascending aorta ratio is greater than 1.0 cm, which correlates with high PA pressure and is suggestive of pulmonary hypertension. CT, computed tomography; MPA, main pulmonary artery; PA, pulmonary artery; SSc, systemic sclerosis.

**
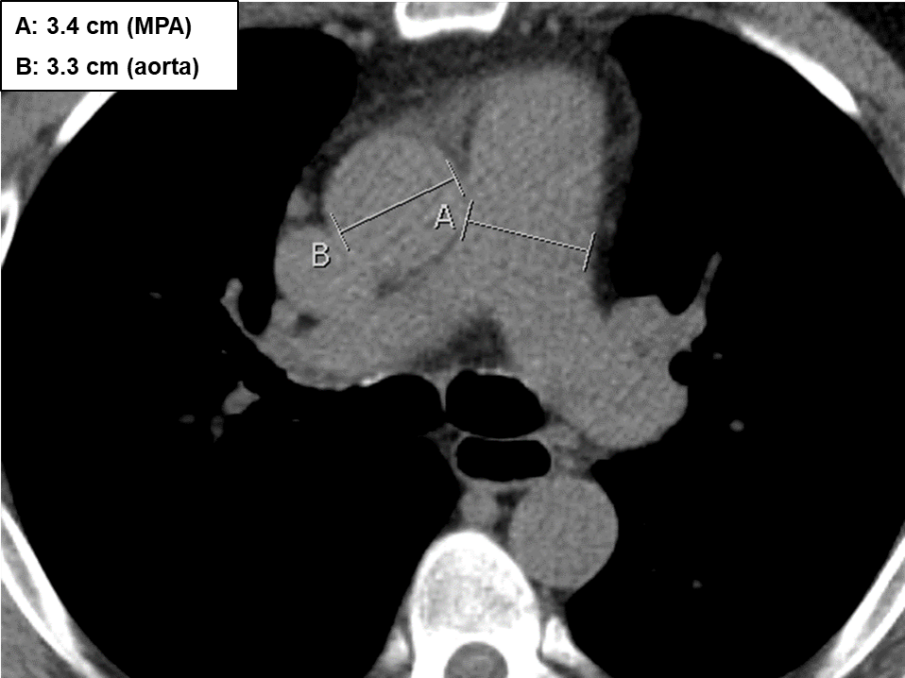
**

**Case study:** A case study of a patient with systemic sclerosis seen at Cleveland Clinic, Ohio.

A 46-year-old male, an ex-smoker, was initially seen in our rheumatology clinic in January 2018. He had been doing well until March 2017, when he developed puffiness and tightness of the skin of his hands and feet. He also developed diffuse joint pains with prolonged morning stiffness and a limited range of motion in his joints. In the next few months, contractures developed in his fingers, wrists, elbows, shoulders, and ankles. Skin tightness progressively affected his fingers, hands, forearms, upper arms, face, and feet. His modified Rodnan skin score (mRSS) was 35/51. In August 2017, he developed multiple ischemic digital ulcers and digital pitting scars. As it started getting colder in October 2017, Raynaud phenomenon became noticeable.

Initial investigations:

- Complete blood count: White blood cell count = 14.17 k/µL (absolute neutrophil count = 11.35 k/µL, 80%); hemoglobin = 10.7 g/dL; MCV = 83.2 fL; platelet count = 694 k/µL.
- Comprehensive metabolic panel: Albumin = 3.3 g/dL; sodium = 127 mmol/L; chloride = 87 mmol/L; creatinine = 0.87 mg/dL.
- Antinuclear antibody (ANA) by indirect fluorescent antibody (IFA) assay (>1:1280, homogeneous pattern); positive anti-topoisomerase-1 (anti-Scl-70) antibody.
- Creatine kinase = 311 U/L (reference range: 42–196 U/L); aldolase = 8.5 U/L (reference range: 1.2 – 7.6 U/L).
- Pulmonary function tests: forced vital capacity = 69% predicted; diffusion capacity of the lungs for carbon monoxide = 48% predicted.
- Thoracic high-resolution computed tomography (CT) scan (December 2017): Lower lobe predominant diffuse ground-glass opacities with associated reticulation and mild traction bronchiectasis (Supplementary Figure 5a and 5b). No evidence of honeycombing or sub-pleural sparing. Fluid-filled and patulous esophagus. There were mild diffuse esophageal dilation and mild mediastinal lymphadenopathy.
- The echocardiogram was normal, with no evidence of pulmonary hypertension.

This constellation of clinical manifestations, including Raynaud phenomenon, puffiness, and tightness of the skin of hands and feet, sclerodactyly and proximal scleroderma, multiple digital pits and ulcers, interstitial lung disease, and polyarthritis, along with positive ANA and anti-Scl-70 antibody confirmed the diagnosis of diffuse cutaneous systemic sclerosis (dcSSc).

In July 2017, he was started on methotrexate 10 mg weekly and prednisone 15 mg daily by a local rheumatologist. As his skin tightness progressed and joint pains worsened, a month later, the dose of methotrexate was increased to 20 mg weekly. In September 2017, he started developing exertional dyspnea, a dry cough, acid reflux (heartburn), and progressive proximal muscle weakness. He lost about 30 lbs., attributed mainly to loss of appetite. He also noticed a lack of libido and erectile dysfunction. In November 2017, his exertional dyspnea and dry cough worsened. Methotrexate was discontinued, and he was started on oral cyclophosphamide 50 mg daily.

He was first seen in our rheumatology clinic in January 2018. We offer a multidisciplinary approach to the management of patients with systemic sclerosis, comprising experts from rheumatology, pulmonology, gastroenterology, cardiology, nephrology, and dermatology. He was started on duloxetine and gabapentin for his severe chronic pain. Omeprazole 40 mg daily was continued for his acid reflux disease. Our pulmonologist optimized his cyclophosphamide dose to 150 mg daily (2 mg/kg), along with low-dose prednisone (20 mg daily tapered over five months) and pneumocystis prophylaxis (trimethoprim-sulfamethoxazole double-strength, three times weekly). He was placed on nebulized hypertonic saline with albuterol for his cough with mucus hypersecretion.

Our gastroenterologist saw him for his severe gastrointestinal symptoms (acid reflux disease, esophageal dysphagia, malabsorption, severe progressive weight loss). Esophagogastroduodenoscopy showed dilation in the entire esophagus, decreased esophageal motility, and an open distal lower esophageal sphincter, consistent with systemic sclerosis. There was LA Grade B reflux esophagitis. He also had mild gastric antral vascular ectasia without bleeding. The antral biopsy was negative for *H. pylori*. The pylorus was wide open, and bilious fluid was found on the greater curvature of the stomach. A gastric emptying study revealed 11-20% gastric retention at four hours, consistent with mild gastroparesis.

On his follow-up visit in April 2018, the patient was found to have lost more weight. He continued to have a non-productive cough causing disturbed sleep. His mobility became progressively limited due to joint pains, muscle wasting and weakness, and shortness of breath on exertion. Prednisone did not lead to any noticeable improvement in his breathing. In addition, he had been having frequent bouts of diarrhea.

In May 2018, as cyclophosphamide was ineffective in treating his interstitial lung disease and progressive skin tightening, he was switched to mycophenolate mofetil 3000 mg daily. A chest CT in May 2018 demonstrated new airspace consolidation in the superior segment of the right lower lobe and posterior-basal segments of bilateral lower lobes, with distribution favoring aspiration (Supplementary Figures 6a and 6b).

In June 2018, he developed more pronounced stiffness in his upper body. His fatigue had increased dramatically, and he had developed increased stiffness in his legs. His shortness of breath had worsened. In August 2018, a chest CT showed diffuse airspace consolidation in the right lower lobe with multifocal nodular consolidations in the right middle and upper lobes with associated bronchiolitis suggestive of bronchopneumonia (Supplementary Figures 7a and 7b). Aspiration pneumonia was considered in the setting of a dilated and contrast-filled esophagus. Preferential right lung involvement may have been related to preferred right-side-down sleeping. Pleural effusions were larger.

A repeat upper endoscopy in August 2018 showed LA Grade A esophagitis and a 2-cm hiatal hernia. In addition, esophageal mucosal changes were suspicious for long-segment Barrett's esophagus. A 10 French nasojejunal Corpak feeding tube was inserted into the jejunum. After some initial struggles with the formula, he started tolerating his feeds well. However, weight loss continued. Long-term enteral feeding access was discussed, and a laparoscopic jejunostomy was planned.

The patient remained quite limited with his underlying systemic sclerosis and overall deconditioning. He started using a wheelchair. He continued mycophenolate 3000 mg daily (liquid). In addition, he was started on monthly high-dose intravenous immunoglobulin infusions (2 grams/kg/cycle) to address his rapidly progressive skin tightness and severe musculoskeletal symptoms. He tolerated his first cycle well.

On September 16, 2018, his Corpak feeding tube became blocked, and he could not get any formula in. He went to the emergency room, where the blockage was cleaned out, and he was discharged. Unfortunately, the patient died in his sleep the following morning. The family did not consent to an autopsy.

**Supplementary Figure 5**

**a b**


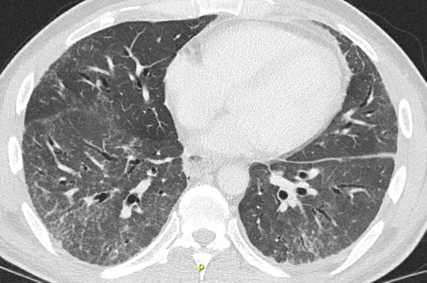

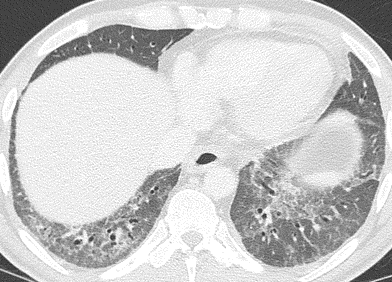


**Supplementary Figure 6**

**a b**


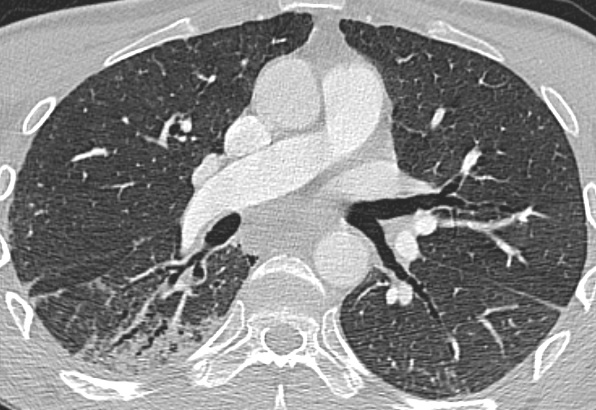

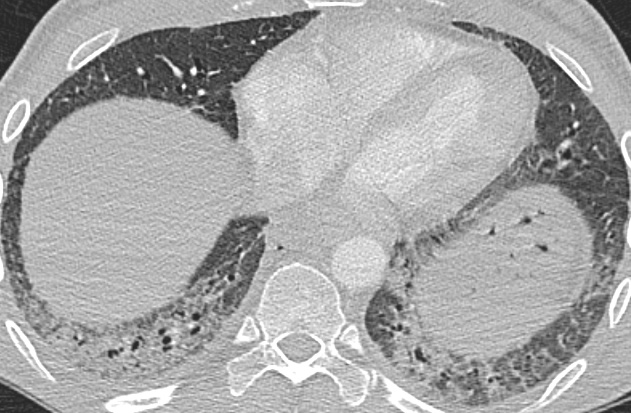


**Supplementary Figure 7**

**a b**


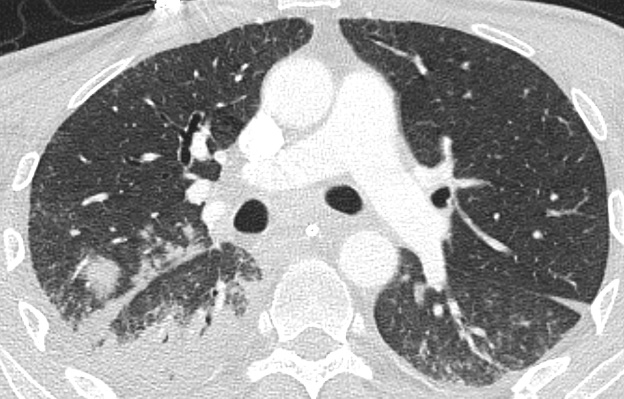

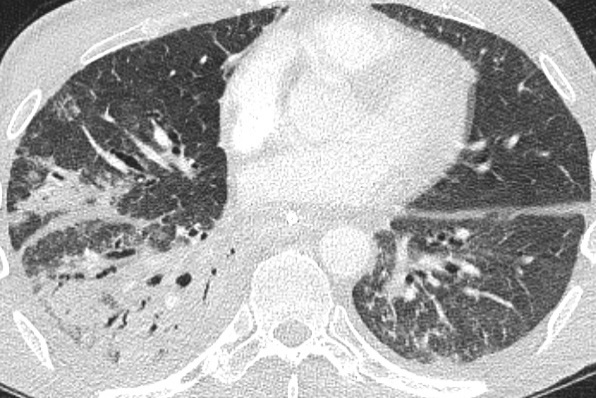

Supplement: Supplementary file 1 — Supplementary file1 (DOCX 2.20 mb) [file 10067_2022_6408_MOESM1_ESM.docx]
